# Assessing the topographic distribution of legacy soil phosphorus in agricultural fields of the Delmarva Peninsula, Mid‐Atlantic Coastal Plain, USA

**DOI:** 10.1002/jeq2.70101

**Published:** 2025-11-29

**Authors:** Maryam Foroughi, Ling Du, Isis S. P. C. Scott, W. Dean Hively, Zachary P. Simpson, Zacharias J. Smith, Cathleen J. Hapeman, Martin C. Rabenhorst, Raymond R. Weil, Gregory W. McCarty

**Affiliations:** ^1^ Department of Environmental Science & Technology University of Maryland (UMD) College Park Maryland USA; ^2^ Department of Biological and Agricultural Engineering Kansas State University Manhattan Kansas USA; ^3^ U.S. Geological Survey (USGS) Lower Mississippi‐Gulf Water Science Center Beltsville Maryland USA; ^4^ USDA Sustainable Water Management Research Unit Stoneville Mississippi USA; ^5^ USDA‐ARS Hydrology and Remote Sensing Laboratory, Beltsville Agricultural Research Center Beltsville Maryland USA

## Abstract

Phosphorus (P) management remains a challenge in agricultural watersheds. The Choptank River Conservation Effects Assessment Project watershed, located in Maryland and Delaware and draining to the Chesapeake Bay, contains legacy soil P from historical dairy and poultry manure applications. These practices elevated soil P beyond crop needs, contributing to persistent P export to aquatic ecosystems. We assessed spatial P distribution and analyzed GIS (Geographic Information Systems)‐derived landscape features driving legacy P movement on a farm (47 ha). We hypothesized that P accumulates in drained lowlands and depressional areas due to gravity‐driven processes that accelerate P‐enriched water to receiving waters via overland flow. In collaboration with the US Department of Agriculture Legacy P project, we collected 105 soil samples (0‐ to 5‐cm and 5‐ to 15‐cm depths) and 14 ditch sediment samples across five topographic openness classes from a farm with >100 years of dairy manure application. Average Mehlich‐III P concentrations were 218 and 179 mg kg^−1^ at 0‐ to 5‐cm and 5‐ to 15‐cm depths, respectively, with legacy areas defined by P content > 100 mg kg^−1^. Soil P and clay particle size were positively correlated (*r* = 0.42, *p* < 0.05), increased as landscape openness decreased, and were negatively correlated with topographic openness (ranging from −0.2 to −0.4, *p* < 0.05), indicating accumulation of P and clay in low‐lying areas. These patterns suggest that historical field‐level managements have primarily shaped P distribution, while hydrologic and landscape properties further influence its redistribution via transport pathways and drainage. These findings support the development of landscape models to map critical source areas in low‐relief watersheds and guide targeted mitigation in high‐risk P export zones.

## Core Ideas

- Topographic metrics, tied to water flow and landscape position, strongly correlated with soil P distribution.
- Soil P links to water flow and accumulation indicators (topographic wetness index [TWI], flow accumulation [FA], and catchment area [CA]), showing hydrology's role in P distribution.
- Correlating topographic metrics with soil P helps identify critical areas for P loss in agricultural landscapes.
- Topographic metrics predicting soil P distribution can be used in precision agriculture and watershed management.

## Plain Language Summary

Phosphorus (P) management is a key issue in agricultural watersheds, such as the Choptank River in Maryland and Delaware, where historical dairy and poultry manure applications have left soils with “legacy P” far exceeding crop needs. These soils are major sources of ongoing P pollution in Chesapeake Bay and other waterways. We examined soil P distribution and how landscape features affect its movement. Lowlands and depressions tend to accumulate P due to natural processes, and water carries it from there to streams via surface or tile drainage. Historical land use and engineered drainage systems have influenced P distribution and transport. Soil samples revealed the highest P levels in areas with high clay content, while open areas (like ridge or hill crest) had lower P levels likely due to soil erosion caused by tillage and water movement. These insights can help create maps to pinpoint critical source areas and guide the placement of mitigation practices for better water quality management.

**Abbreviations**
CA: catchment area
CSA: critical source area
DEM: digital elevation model
DPS_ox_
: degree of oxalate P saturation
FA: flow accumulation
GCur: general curvature
LS: slope length factor
M3‐P: Mehlich‐III P
MAE: mean absolute error
PCur: profile curvature
PlCur: plan curvature
POP: positive openness
RePC1: topographic relief component 1
RePC2: topographic relief component 2
RF: random forest
RMSE: root mean squared error
TWI: topographic wetness index
UpSl: upslope slope
USDA: United States Department of Agriculture

## 1 INTRODUCTION

Nutrient legacy issues, particularly concerning phosphorus (P) and nitrogen (N), pose significant challenges for water quality management and watershed mitigation efforts (Paytan & McLaughlin, 2007). Phosphorus and N movement from land into aquatic systems is a major driver of eutrophication, which is the leading cause of surface water impairment in the United States (Chang et al., 2021; Paerl et al., 2014; Paytan & McLaughlin, 2007). This issue is particularly acute in Chesapeake Bay, where excessive nutrient inputs have led to algal blooms and hypoxia conditions (Kleinman et al., 2019), and where agriculture is a significant contributor to P and N enrichment (Ator et al., 2019; Carpenter et al., 1998; Howarth et al., 2002).

Although Chesapeake Bay presents a key case study of nutrient legacy challenges, the concept of critical source area (CSA) has an established global history and has been implemented in Europe, Oceania, and Asia to target nutrient and sediment reduction. These international applications highlight the adaptability and effectiveness of CSA‐based management beyond North America. Since the 1990s, CSA research has emphasized phosphorus, building on the foundational work of Andrew Sharpley (A. N. Sharpley, 1985, 2011; A. Sharpley et al., 1998), whose contributions helped integrate science and nutrient policy to improve water quality (McDowell et al., 2024).

Building on this global context, Chesapeake Bay remains one of the most intensively studied watersheds in the United States, and nutrient pollution continues to challenge environmental policy and land management. In 2021, tributaries delivered approximately 9.03 million kg of P and 130 million kg of N to the Bay (Chesapeake Bay Program, 2025). Modeling indicates agriculture accounts for 56% of this total P load (Chesapeake Bay Program, 2018). Mitigation of nonpoint‐source pollution requires addressing both particulate and dissolved P losses from agriculture (Kleinman et al., 2019). Fertilizer and manure applied to farmland are primary P sources to the Bay (Sims et al., 2002). Legacy P accumulates in soils and is slowly released (A. Sharpley et al., 2013), whereas legacy N is more mobile and can persist in groundwater for decades (Van Meter et al., 2016). Addressing these legacy nutrients requires an integrated approach ensuring efforts to mitigate legacy P losses with strategies for managing legacy N. This alignment is essential to achieving long‐term water quality goals while identifying CSA (A. N. Sharpley et al., 2011).

The Delmarva Peninsula is located in the Mid‐Atlantic region of the United States and encompasses most of Delaware, Maryland east of Chesapeake Bay, and a portion of Virginia. The Delmarva Peninsula contains small depressional wetlands, known as Delmarva bays, which are interspersed throughout agricultural landscapes. Since 2014, approximately 41% of these wetlands have been completely converted to agriculture, and another 29% have been partially drained with areas of lower relief and natural drainage more likely to be prioritized for conversion due to shorter hydroperiods associated with their advanced public ditch networks (Fenstermacher et al., 2014). Engineered drainage systems, such as rotary ditches created within fields and public ditch networks, have altered hydrologic connectivity, enhancing nutrient transport across the landscape (Carter, 2015; Jones et al., 2018; McCarty et al., 2008). These ditches draining nutrient‐enriched soils, regularly treated in poultry litter, enhance the likelihood of nutrient transport to surface waters (Mozaffari & Sims, 1994). On the Delmarva Peninsula, the number of broiler chickens produced steadily increased from 100 million birds in the late 1940s to approximately 600 million in the late 1990s where it remains today (Delmarva Chicken Association, 2020). Conversely, the number of dairies has decreased from over 5600 small‐scale dairy farms in 1930s (United States Bureau of the Census, 1932) to less than 300 larger farms in 2023, due in part to industry consolidation (Cribbs, 2024). Before 1990s, when manure testing and evidence‐based management became common, excessive application of dairy manure and poultry litter caused P to accumulate in agricultural soils, leading to sustained P loss to the Chesapeake Bay (Patterson et al., 1998; Vadas & Sims, 1998). While these regional drainage studies document hydrologic alteration, few have quantified how these systems interact with long‐term P legacies at the field scale.

Legacy P stored in soils and sediments from CSA can continue to impact water qualities despite reductions in current P applications (Kleinman et al., 2019; Simpson et al., 2024). While N legacies also persist, this study focuses on P due to its stronger spatial associations with topographic and hydrologic features in the Delmarva region. Legacy P often leaches into drainage networks via preferential and lateral flow paths, degrading water quality and complicating mitigation efforts (Mozaffari & Sims, 1994; Sallade & Sims, 1997; Toor & Sims, 2015). Managing P losses from large legacy pools is crucial because the depletion of soil P by crop harvest removals may take decades (Fiorellino et al., 2017). In the short term, targeted interventions can help to mitigate legacy P impacts, which is the goal of the US Department of Agriculture (USDA) Legacy Phosphorus Study (Qin et al., 2018; A. Sharpley et al., 2013; Simpson et al., 2024).

**Core Ideas**

- Topographic metrics, tied to water flow and landscape position, strongly correlated with soil P distribution.
- Soil P links to water flow and accumulation indicators (topographic wetness index [TWI], flow accumulation [FA], and catchment area [CA]), showing hydrology's role in P distribution.
- Correlating topographic metrics with soil P helps identify critical areas for P loss in agricultural landscapes.
- Topographic metrics predicting soil P distribution can be used in precision agriculture and watershed management.

High‐resolution soil P maps based on soil testing can be impractical to make at large scales due to logistical and financial constraints. While fine‐scale prediction using pH, texture, and organic matter is effective (e.g., Hosseini et al., 2017), such detailed data are not typically available over broader regions. At larger scales, additional factors like climate, weather, geology, and topography become increasingly influential. Topography influences processes such as soil formation, runoff, tillage, and water erosion, which drive P redistribution across landscapes (Roberts et al., 1985). For instance, soil P concentrations often decrease with increasing slope gradients due to the gravity‐driven transport of soil and nutrients (He et al., 1995). Advances in geospatial data availability and machine learning offer new opportunities to model complex environmental patterns, including soil P distributions, across diverse landscapes (Zhong et al., 2021). Remote sensing enables large‐scale monitoring of soil and vegetation properties, supporting digital soil mapping and improving sediment and nutrient management (Du et al., 2024; Kubiak et al., 2024; Selmy et al., 2025). Machine learning approaches, such as random forests (RFs) based on detailed geospatial data, have been effectively used to predict soil P at local scales (Jeong et al., 2017; Kaya & Başayığıt, 2022; Matos‐Moreira et al., 2017).

Traditional geostatistical methods (e.g., kriging and interpolation) have provided insights into soil nutrient distributions but often fail to account for complex relationships between soil properties and environmental variables (Guo et al., 2024; Zhang et al., 2007). Machine learning methods, which can capture nonlinear interactions and handle diverse datasets, are increasingly being applied to improve digital soil mapping (Hengl et al., 2015; Yan et al., 2022). However, these approaches have not been extensively applied to estimate soil total and available P across the Delmarva Peninsula, particularly in the Choptank River watershed, leaving important questions about drainage‐driven P export unaddressed.

This study investigated how topographic metrics influence soil P distribution and predicted CSA in the Choptank River watershed. We hypothesized that soil P is linked to terrain features associated with water accumulation and flow, such as slope length and depressional areas, due to sediment transport and historical land management practices. Although regional studies have documented hydrologic modifications, few have applied spatial modeling to quantify the relationship between landscape topography and soil P concentrations at the field scale. By integrating topography, management practice, and soil characteristics, this research investigates and offers new insights into legacy P dynamics and site‐specific strategies for precision conservation. The use of RF modeling introduces a novel method for enhancing soil nutrient management and addresses important gaps in existing research.

## 2 MATERIALS AND METHODS

### 2.1 Study site

**FIGURE 1:**
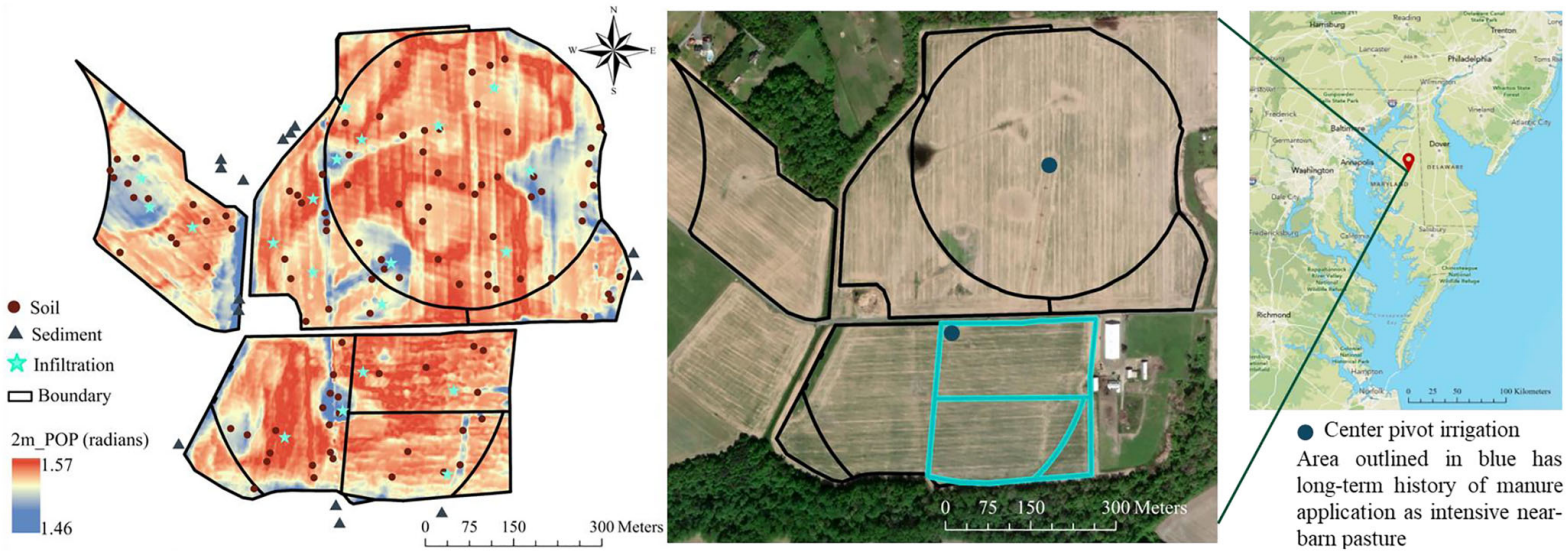
Geographic location of the field study. (Left) A map showing the positive openness (POP) used to select soil, sediment, and infiltration sample location. (Right) A map of the study site highlighting the area that had a long‐term history of manure application as an intensive near‐barn pasture. Basemap and imagery sources include Earth Community Maps Contributors, Esri, OpenStreetMap, USGS, and other contributors via ArcGIS Pro.

The study farm is located in the Tuckahoe Creek watershed (a tributary of the Choptank River) with sampled fields totaling 47 ha (Figure 1). This area had an average annual precipitation of 1180 mm and a mean annual temperature of 14.4°C between 1991 and 2020 (PRISM Climate Group, 2020). The soils on this farm occur in map units named for Hambrook (well drained), Woodstown (moderately well drained), Fallsington (poorly drained), and Corsica (very poorly drained) catena (fine‐loamy, siliceous, mesic Aquic and Typic Hapludults, Endoaquults, and Umbraquults), with better drained soils typically in upland areas and more poorly drained soils in lowland areas (Soil Survey Staff, Natural Resources Conservation Service, United States Department of Agriculture, 2019).

The farm was a 40–100 cow dairy operation for over 100 years, during which soils received continued manure inputs. Part of this farm was previously used as a near‐barn loafing area of perennial pasture receiving considerable manure from intensive animal use (near‐barn pasture) and then converted to annual crops in 2012 (blue outline in Figure 1; land manager, oral communication, 2020). The rest of the farm was historically cultivated with corn, soybean, and wheat with occasional extensive grazing. Since 2018, the farm has been planted to perennial rhizomatous grass (*Miscanthus × giganteus J.M. Greef & Deuter*). Due to the high‐water table, the land manager implemented rotary ditches (a type of shallow surface drain) and open inlet tile drains to enhance water drainage in this field.

### 2.2 Sample collection

To characterize terrain features, 14 topographic covariates were generated using the System for Automated Geoscientific Analysis v. 7.3.0, derived from a 2‐m‐resolution digital elevation model (DEM) obtained from lidar data from 2003, 2006, and 2007 in Maryland (Du et al., 2020). Covariates included positive openness (POP), upslope slope (UpSl), profile curvature (PCur), plan curvature (PlCur), general curvature (GCur), flow accumulation (FA), topographic relief components 1 and 2 (RePC1, RePC2), flow path length, catchment area (CA), topographic wetness index (TWI), stream power index, and slope length factor (LS). RePC1 exhibits a strong correlation with coarse resolution of relief variation. In contrast, RePC2 shows a high correlation with fine resolution and is associated with small‐scale variations in relief (X. Li et al., 2018).

Soil sampling locations were determined by using terrain attributes derived from elevation, including topographic openness, which measures angles between a point and surrounding terrain, and higher values indicate convex features like ridges or crests. To provide sufficient data, 105 soil samples were collected across five topographic openness classes to capture the P concentration variability. The classes were defined by POP radiance values: <1.555, 1.55500001–1.559, 1.59900001–1.563, 1.56300001–1.567, and >1.567. Within each class, 21 sampling points were randomly selected (Figure 1). Samples were collected in spring 2022, using a 3.04‐cm diameter soil probe from 105 locations. At each location, four subsamples were taken and divided into 0‐ to 5‐cm and 5‐ to 15‐cm depths and composited by depth to form one sample per depth per location. Sediment samples were collected from 14 locations in ditches around the farm. All samples were immediately stored on ice in the dark. Half of each sample was air‐dried, while the other half was frozen at −20°C to preserve it for sensitive analyses such as acid ammonium oxalate extraction (Simpson et al., 2024). Infiltration rates were measured at 20 locations using sprinkler infiltrometers (Van Es & Schindelbeck, 2003) selected via stratified sampling based on soil type and POP map.

### 2.3 Sample analyses

All soil samples were ground to 2 mm and analyzed for standard fertility parameters. Soil physicochemical characteristics, including particle size, total carbon (C), P pools (total P, Mehlich‐III P [M3‐P], and oxalic acid P), total iron (Fe), aluminum (Al), and calcium (Ca), plus oxalic acid extractable Fe, Al, and Ca were analyzed. Mehlich‐III extractions (Mehlich, 1978) determined labile P, Fe, Al, and Ca, while total elements were measured using the digestion method (EPA, 2017). Oxalic acid extraction (McKeague & Day, 1966) was used to extract P, Fe, Al, and Ca in frozen samples (Simpson et al., 2024). Soil total C was determined by the pyrolysis method (ISO, 2024). Soil analyses were conducted at the Penn State Agricultural Analytical Services Laboratory, University Park, PA (https://agsci.psu.edu/aasl). Particle size fractions were measured using a PARIO Plus laser soil particle analyzer (PARIO Plus, METER Group), with particle class boundaries defined by USDA soil texture classification standards. Clay, silt, and sand refer to particle size fractions (%), not mineralogical composition.

### 2.4 Correlation analysis

Correlation analyses were conducted to examine relationships between 14 topographic metrics and total soil P and M3‐P, as well as between soil P (total and M3‐P) and soil parameters including particle size, pH, and total C. Also, the correlations between infiltration landscape metrics, soil parameters, and infiltration were determined using Pearson correlation. Cluster correlation analysis in R software v4.0.2 (R Core Team, 2020) was used to group variables with similar correlation patterns to visualize relationship in the study area.

### 2.5 Topographic model establishment and extrapolation

RF regression was used to determine the relationship of soil total P and M3‐P with topographic metric, field boundary, and soil characteristics at 105 sample locations. Predictions were generated by averaging the outputs of all regression decision trees (Breiman, 2000). RF regression was selected because it is a nonparametric method enhancement of Classification and Regression Tree systems aimed at improving prediction accuracy. RFs consist of multiple regression tree models, where each tree is constructed based on a randomly generated vector sampled independently but sharing the same distribution across all trees in the forest (Breiman, 2000). Data analysis and visualization were conducted using the R programming language v4.0.2 (R Core Team, 2020) and the open‐source “Random Forest” package in R (Liaw & Wiener, 2018). We included 500 trees and employed a leave‐one‐out cross‐validation method. Variable importance was determined using the “importance” function from the “Random Forest” package (Liaw & Wiener, 2002), which quantifies each variable's impact on the model's predictive accuracy in regression analyses. Model performance was evaluated using root mean squared error (RMSE), coefficient of determination (*R* ^2^), and mean absolute error (MAE) (G. Li et al., 2020).

(1) $$\mathrm{RMSE}=\sqrt{\frac{1}{n}\sum_{i=1}^{n}{\left(Pi-Oi\right)}^{2}}$$

(2) $$\mathrm{MAE}=\frac{1}{n}\sum_{i=1}^{n}\left|Pi-Oi\right|$$

where *P_i_* is the predicted value and *O_i_* is the observed value, and *n* is the number of samples with i = 1,2,…,*n*.

To compare total P and M3‐P content between CAs and the rest of research field, we identified CAs using DEM data and ArcGIS‐Pro (Esri, Inc., v 3.3.1).

## 3 RESULTS AND DISCUSSION

### 3.1 Soil nutrient concentrations

**FIGURE 2:**
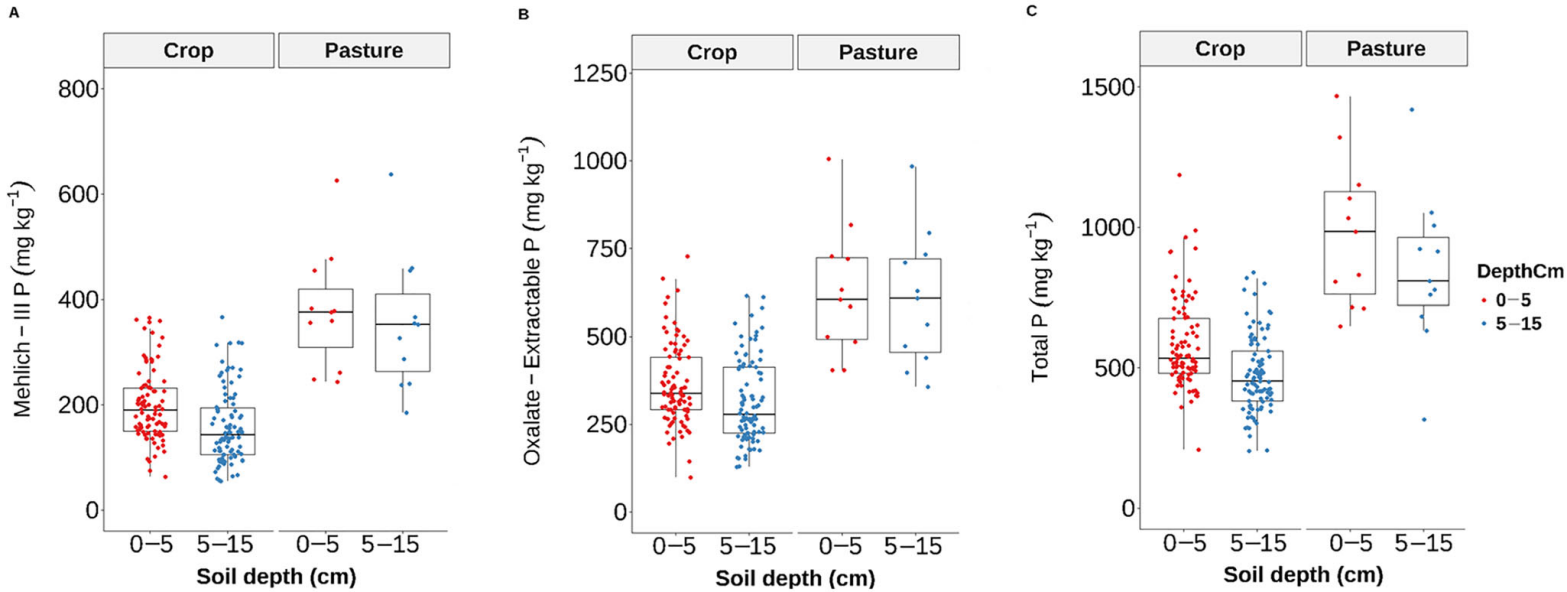
Effect of land use and soil depth on various P pools (mean ± standard deviation [SD], mg kg^−1^) at two different soil depths (0–5 cm [red] and 5–15 cm [blue]). The pasture indicated that the field area had a long‐term history of intensive manure application associated with near‐barn use.

The mean (±standard deviation) concentration of total P was 623 ± 210 mg kg^−1^ and 515 ± 193 mg kg^−1^ at 0‐ to 5‐cm and 5‐ to 15‐cm depths, respectively. The M3‐P averaged 219 ± 92 mg kg^−1^ at 0–5 cm and 179 ± 98 mg kg^−1^ at 5–15 cm (Table S1). These concentrations exceed recommended P soil test thresholds: >100 mg kg^−1^ is considered high, and >150 mg kg^−1^ requires controlled P management in Maryland and Delaware (Sims et al., 2002), posing a high risk to water quality through elevated P concentration in runoff water and eutrophication potential. This underscores the need for improved P management practices in agricultural systems. Our spatial‐RF framework helps identify high‐risk areas for targeted interventions, such as buffer strip placement and drainage modifications. The elevated legacy P concentrations in this field can be attributed to historical dairy and poultry manure applications, contributing to persistently high soil P concentrations that pose challenges for current management in reducing P losses to surface and groundwater systems. Soil total P and M3‐P concentrations in the historical near‐barn pasture area were 68% and 90% higher, respectively, than in the cropland area without grazing history at the 0‐ to 5‐cm depth (Figure 2). This difference likely reflects long‐term manure applications associated with livestock housing and barn cleaning practice, which facilitated P accumulation in the soil. Repeated manure applications have been known to elevate soil P concentrations over time, forming legacy P (Haygarth et al., 2014; Jarvie et al., 2013).

Sediment concentrations of total P (636 ± 390 mg kg^−1^), Al, and total C were higher than in the surface soil. Differences in Al and total C were statistically significant (*p* < 0.05), whereas total P was not (Table S1). This sediment enrichment is likely P in eroded soil and runoff, water transported from upland to low‐lying areas. Phosphorus transport through subsurface pathways is typical in watersheds with high soil P concentrations, low P sorption capacities, and artificial drainage systems such as tile drains or ditches (Smith et al., 1998). In contrast, M3‐P concentrations in sediment were significantly lower than in the soil (*p* < 0.05, Table 1 and Table S1), likely due to sedimentary P leaching into flowing surface water and/or the higher clay concentration in sediment resulting in increased P binding and occlusion. Near the sediment‐water interface, P concentrations decline rapidly due to Al and oxidized Fe binding sites. Consequently, P becomes immobilized through oxidative precipitation, with ferric phosphate formation playing a key role in this process (Baken et al., 2015).

**TABLE 1:** Average of soil total P (mg kg^−1^), total C (g kg^−1^), and soil particle size at 0‐ to 5‐cm soil depth over all the field and catchment areas.

	Total P	M3‐P	Total C	Clay%	Silt%	Sand%
Overall average	623	218	15.4	10.3	33.2	56.0
**Catchment**						
C1	559	**240**	12.4	8.16	27.3	64.5
C2	**647**	196	14.2	10.2	37.3	51.0
C3	**630**	190	16.9	**14.3**	40.2	45.6
C4	539	159	13.1	**10.7**	36.0	53.4
C5	570	**269**	14.3	8.09	17.6	74.3
C6	**1110**	**419**	17.3	**12.2**	41.8	41.0
C7	**648**	**233**	17.0	**10.9**	37.3	51.8
C8	553	172	13.6	6.99	30.4	62.6
C9	**639**	**313**	12.6	6.25	20.2	73.5
**Sediment**	**636**	**113**	**44.0**	**20.0**	**37.0**	**43.0**

*Note*: Bold text indicates higher than average catchment concentrations.

**FIGURE 3:**
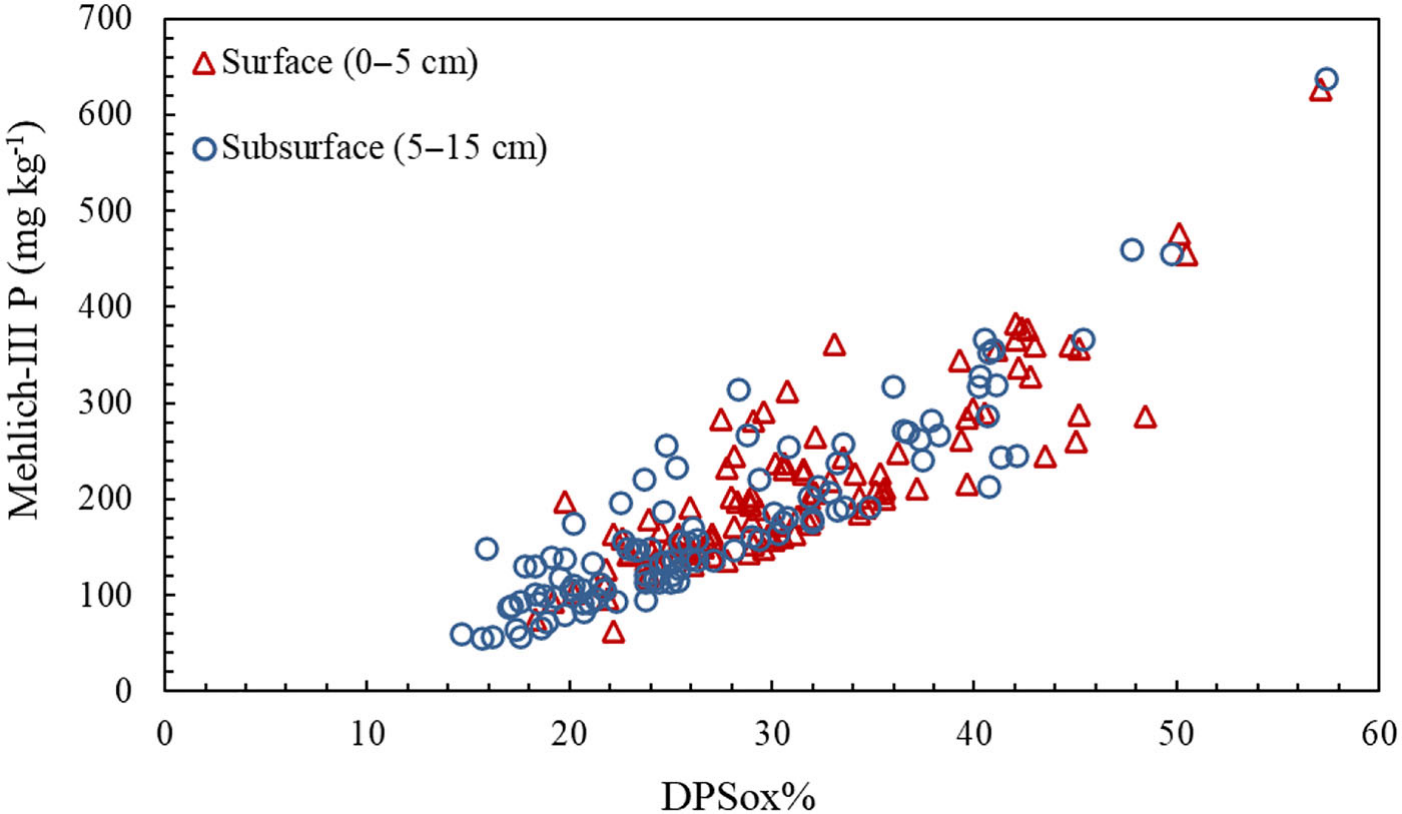
Relationship between soil Mehlich‐III P and percentage of degree of oxalate P saturation (DPS_OX_ %).

The degree of oxalate P saturation (DPS_OX_) showed a linear relationship with M3‐P (Figure 3), averaging 32% at 0–5 cm. Previous studies identified DPS_OX_ values between 25% and 40% as posing environmental risks (Breeuwsma et al., 1995; De Smet et al., 1996). These findings underscore the potential of DPS_OX_ as a reliable indicator of a soil's capacity for nonpoint source pollution (Sims et al., 2002).

### 3.2 Correlation analysis

**FIGURE 4:**
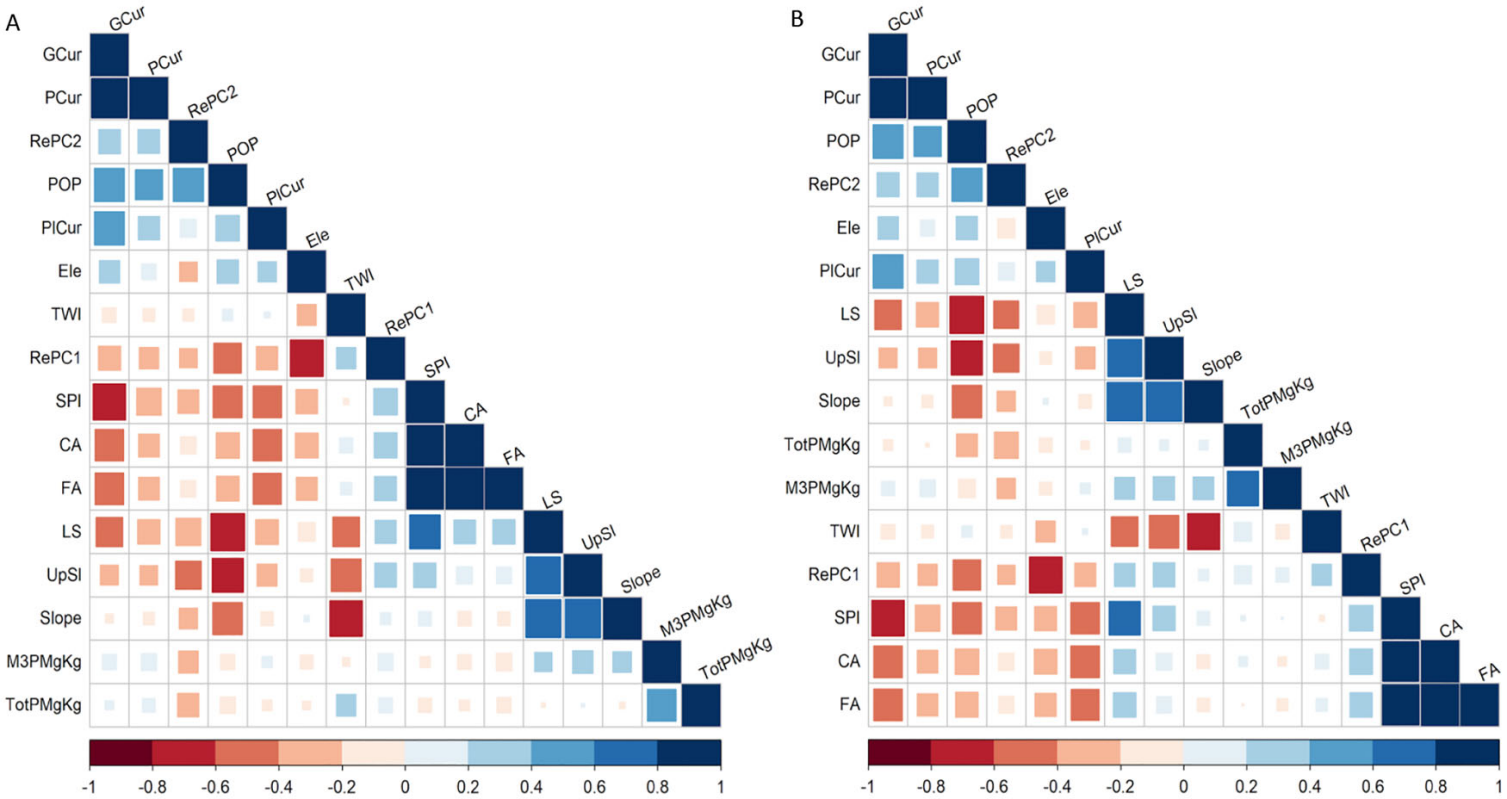
The cluster correlation between topographic parameters and soil P (total P and Mehlich‐III P [M3‐P]) in two soil depths, 0–5 cm (A) and 5–15 cm (B). The topographic metrics include positive openness (POP), upslope slope (UpSl), profile curvature (PCur), plan curvature (PlCur), general curvature (GCur), flow accumulation (FA), topographic relief component 1 (RePC1), topographic relief component 2 (RePC2), flow path length (FPL), catchment area (CA), topographic wetness index (TWI), stream power index (SPI), and slope length factor (LS). Square size represents the magnitude of the Pearson correlation coefficient (|*r*|), with full squares representing |*r*| = 1; color indicates direction (blue = positive, red = negative).

Correlations between topographic parameters and soil P revealed negative relationships between RePC2, POP, and total P and M3‐P in both soil depths (Figure 4 and Figure S1), suggesting P accumulation in depressions via sediment transport. In contrast, a positive correlation between slope, UpSl, and LS versus M3‐P indicates that longer slopes increase erosion potential and water movement, which in turn facilitates P transport. Similarly, the significant positive relationship between TWI and total P suggests that areas with greater water and sediment accumulation are prone to greater P content (Figure S1). The weak correlation between soil P and topographic metrics suggests that land management practices, such as manure application, tillage, and crop rotation, play a significant role. For instance, poorly timed manure applications or reduced tillage in specific areas can cause localized P accumulation, potentially overshadowing topographic effects. Strong correlations between surface and subsurface P (*r* = 0.80 for total P, *r* = 0.96 for M3‐P) suggest limited vertical variability in P distribution due to prior deep tillage (to 20 cm). Recent studies confirm that environmental factors alone do not fully explain soil P variability (Guo et al., 2024).

**FIGURE 5:**
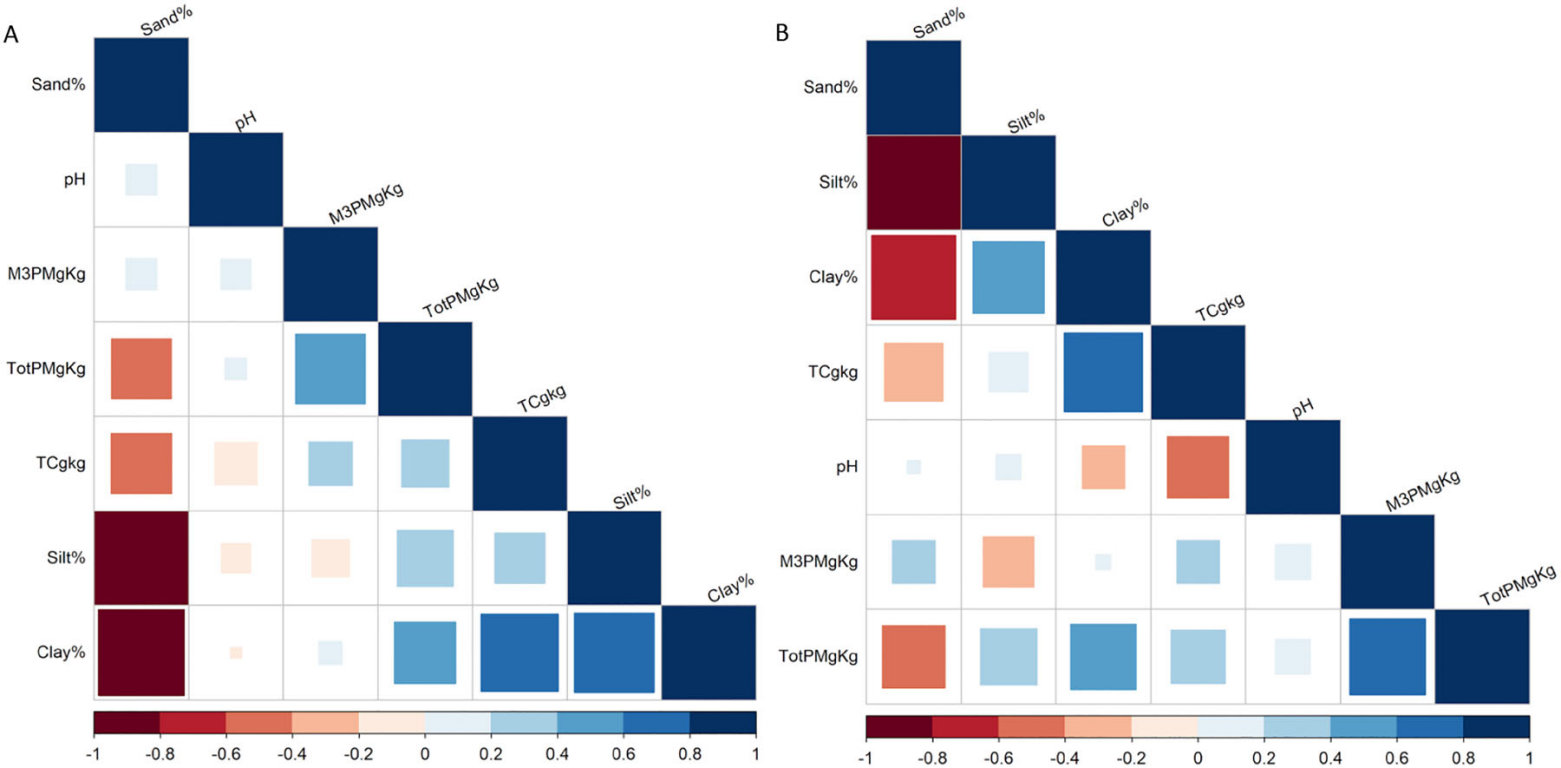
The cluster correlation between soil parameters (sand, silt, clay, pH, and total carbon [TC]) and soil P (total P and Mehlich‐III P [M3 P]) in two soil depths, 0–5 cm (A) and 5–15 cm (B). Square area reflects the magnitude of the Pearson correlation coefficient (|*r*|), and color indicates direction (blue = positive, red = negative), consistent with the visualization approach described in Figure 4.

Regarding soil properties, total P was positively correlated with clay content and negatively correlated with sand content at both soil depths (Figure 5). Clay content showed a strong correlation with Ox‐Fe (*r *= 0.64), consistent with lowland areas where sediment deposition and higher clay content contribute to elevate P concentrations. While Fe oxides stabilize P through sorption, reducing short‐term loss potential, these zones may also accumulate legacy P. Infiltration rates were positively correlated with sand% (*r* = 0.47), POP (*r* = 0.49), GCur (*r* = 0.60), PCur (*r* = 0.61), PlCur (*r *= 0.18), and RePC2 (*r* = 0.38). These relationships suggest that sandy upland soils facilitate water infiltration, limiting P accumulation. In contrast, infiltration was negatively correlated with clay content, slope, UpSl, and RePC1, indicating poor drainage conditions typical of depressional areas (Figure S2). The positive correlation (*r* = 0.23) between clay content and RePC1 and UpSl supports the observation of higher clay concentrations in low‐lying area.

### 3.3 Topographic model establishment and extrapolation

The RF model identified soil texture (specifically clay and sand content), RePC2, soil total C, and field boundary as the most influential predictors of total P and M3‐P at both depths (Figures S2 and S3). The model performance metrics for total P predictions included an RMSE of 177 mg kg^−1^, an *R* ^2^ of 0.29, and an MAE of 131 mg kg^−1^ (Table S2; Figure S3). Previous research studies reported a similar *R* ^2^ value (*R* ^2^ = 0.29) for predicting the available soil P pool, whereas in our study, the *R* ^2^ for M3‐P was 0.44 at 0–5 cm (e.g., Guo et al., 2024). At 5‐ to 15‐cm depth, model performance improved, with *R* ^2^ = 0.47 and RMSE = 100 mg kg^−1^ for total P, and *R* ^2^ = 0.48 with RMSE = 71.8 mg kg^−1^ for M3‐P (Table S2; Figure S4). The RMSE value for total P in this study indicates moderate accuracy, particularly when compared with other studies that reported higher RMSE values for total P prediction. For example, partial least squares and backpropagation neural network models were classified as having excellent prediction accuracy for total P where an RMSE of 1140 mg kg^−1^ was achieved (Reda et al., 2020). In contrast, the relatively high predictive accuracy for total P using an RF model has been demonstrated where the *R* ^2^ was 0.58 and RMSE was 129 mg kg^−1^ for an independent validation dataset (Dolph et al., 2023). While these studies used different approaches and variables, our RF model performance, with an RMSE of 177 mg kg^−1^ and *R* ^2^ = 0.29, is reasonable for exploratory modeling at field‐to‐farm scales. Given our study focus on fine‐scale spatial variability and the integration of topographic metrics and soil properties, this performance supports the utility of RF for identifying relative patterns and CSA rather than precise point‐level predictions.

### 3.4 Additional predictive variables that influence hydrology and connectivity

Using the traditional approaches of soil texture and total C provided a reliable estimate of total P distribution but also highlighted opportunities for further refinement by incorporating additional predictive variables, such as water table depth and hydrologic connectivity. The relationship between observed values and those predicted by the RF model demonstrated strong correspondence for clay content (*R* ^2^ = 0.40, RMSE = 3.12%, MAE = 2.10%) and total carbon (*R* ^2^ = 0.59, RMSE = 2.62 g kg^−1^, MAE = 1.84 g kg^−1^). In contrast, sand content and infiltration showed weaker agreements, with *R* ^2^ values of 0.29 and 0.28, respectively, and higher RMSE values of 11.8% for sand content and 0.20 for infiltration (Table S2; Figure S5). This weak relationship between observation and prediction may indicate underlying field management effects, particularly variable legacy P inputs resulting from practices such as conservation tillage, cover cropping, or reduced P application rates, which can exert a stronger control on soil P distribution than topographic or hydrologic processes (Kleinman et al., 2022). Fields with historically low or high P applications can mask or override topographic signals, reinforcing the need to account for management history when interpreting spatial patterns of nutrient accumulation.

**FIGURE 6:**
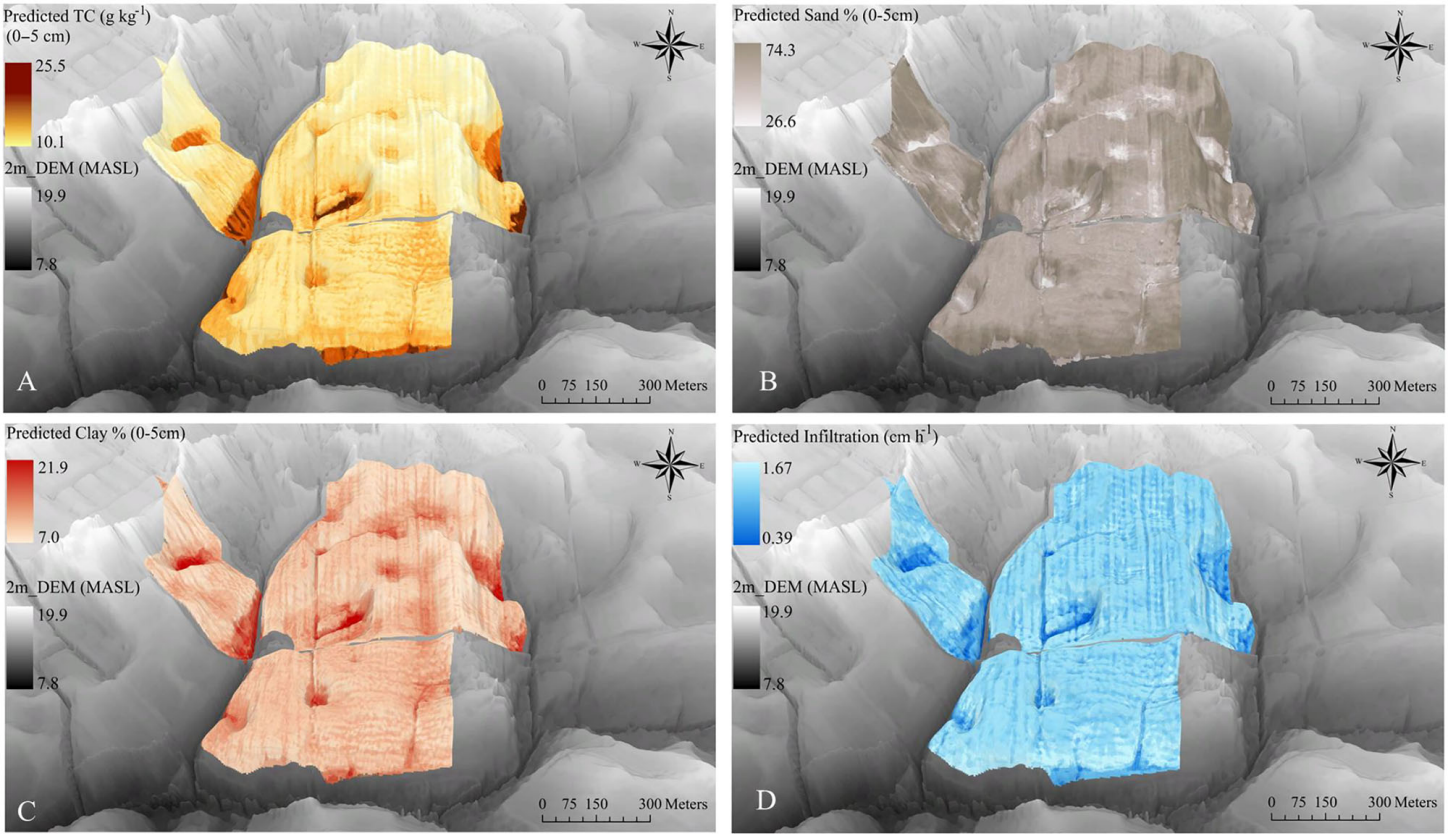
3D predicted maps of soil properties: (A) soil total carbon (TC) (g kg^−1^), (B) sand%, (C) clay%, and (D) infiltration (cm h^−1^) using the Random Forest model. Digital elevation map referenced to the North American Vertical Datum of 1988 (NAVD 88).

**FIGURE 7:**
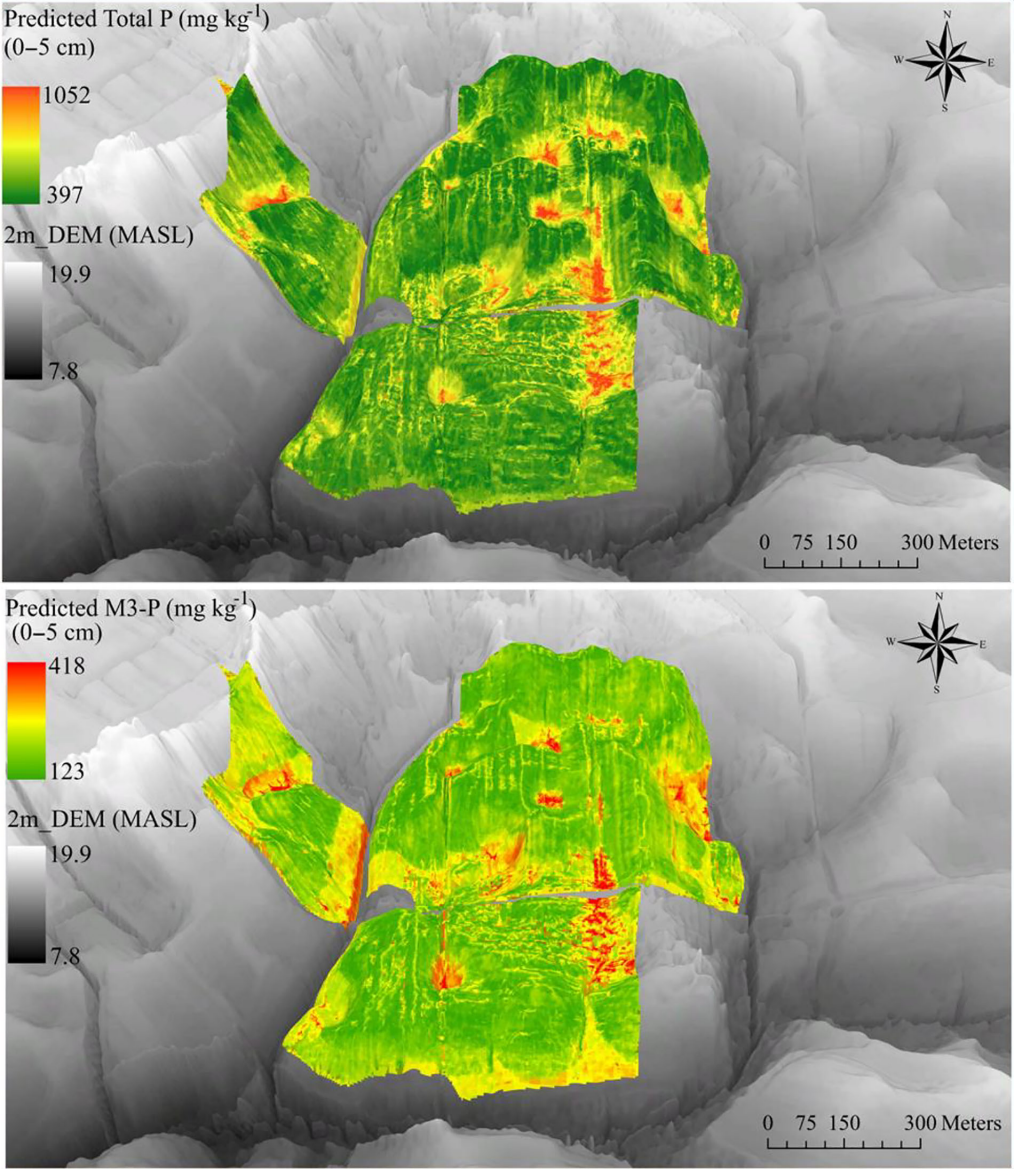
3D predicted maps of total P (mg kg^−1^) and Mehlich‐III P (M3‐P) (mg kg^−1^) at 0‐ to 5‐cm soil depth using the Random Forest model. Digital elevation map referenced to the North American Vertical Datum of 1988 (NAVD 88).

Maps for soil total C and for clay showed higher content in depressional areas (i.e., drained wetland), while the infiltration rates were low in these areas (Figure 6). The RF model was then used to predict P distribution based on topographic metrics, infiltration, soil texture, and total C. For total P predictions, the RMSE was 177 and 100 mg kg^−1^, the *R* ^2^ was 0.29 and 0.47, and the MAE was 132 and 143 mg kg^−1^ for 0‐ to 5‐cm and 5‐ to 15‐cm soil depth, respectively, indicating moderate predictive accuracy. The resulting maps highlighted higher total P concentrations in depressional areas, consistent with their positive correlations with clay and total C (Figure 7). These findings suggest that depressions act as P sinks, accumulating P through sediment deposition and organic matter accumulation as reported previously (Mumbi et al., 2024; Page et al., 2005). Although depressions appear to function as P sinks, their high clay and Fe‐oxide content may enable future P mobilization under reductive conditions, suggesting potential for transition into CSAs depending on environmental triggers (Baken et al., 2015; Chen & Arai, 2023). Despite the moderate predictive accuracy for clay content (*R* ^2^ = 0.40) and total C (*R* ^2^ = 0.59), the RF model's lower *R* ^2^ values for total P (0.29) and M3‐P (0.44) at 0–5 cm suggest that field management practices, such as manure application timing and drainage system engineering, may introduce variability not fully captured by the topographic and soil parameters alone. The inclusion of field boundary as an important variable notably improved model performance for M3‐P. The relatively high RMSE values, particularly for sand content (11.8%) and total P (177 mg kg^−1^), further emphasize the need for additional site‐specific data, such as soil moisture, depth to water table, and historical P application records, to improve model performance. Furthermore, managing these areas to minimize P losses is critical because they can shift from sinks to sources under certain hydrologic conditions. Conversely, sand content was higher in upland areas, which aligns with its negative correlation with total P, likely due to faster infiltration and lower retention capacity (Figure S5).

### 3.5 Influence of depressional areas as CSAs

The model predicted higher total P concentrations in depressional areas, consistent with observed correlations with soil properties. Catchment‐scale analysis showed that mean M3‐P or/and total P levels in depressional areas often exceeded the average values observed across all samples in the field, possibly indicating both preferential accumulation and relative P depletion from upslope areas (Table 1; Figure S6). These areas, often associated with historical near‐barn pasture, also exhibited higher clay content than the overall field average. Although the positive correlations between total P and clay content and soil total C suggest that water transport from uplands to lowlands contributes to P accumulation (Figure S1), measured total P and M3‐P concentrations in depression areas were not significantly higher than in non‐depressions (*p* > 0.05; Table 1). Targeted mitigation strategies, such as constructed wetlands, vegetative buffers, or two‐stage ditches, may help intercept flow and reduce P export risk in these hydrologically sensitive zones, particularly areas with high‐water tables, frequent saturation events, or poorly drained soils. These conditions can mobilize legacy P, shifting such areas from nutrient sinks to sources. Managing these areas to minimize P losses is critical because they can shift from sinks to sources under certain hydrologic conditions. Some depressional areas exhibited lower P concentrations, and the weaker relationship between observed and predicted P values in the RF model may be attributed to engineered drainage systems, such as rotary ditches and tile drains, and surrounding zones with similarly low P levels, possibly resulting from field‐level management history. These systems facilitate surface water export, increase water velocity, and reduce retention time for P accumulation or interaction with soil particles (Mendes, 2020), thereby elevating loss potential even in areas with lower soil P. The study area contains depressional features that have been drained for agricultural use, which constitute Delmarva bays (Fenstermacher et al., 2014). In regions like the Delmarva Peninsula, artificial drainage systems play a significant role in P transport in sandy soils (Sims et al., 1998).

Overall, the performance metrics of the RF model, especially the RMSE and MAE values for total P (177 and 131 mg kg^−1^, respectively), highlight both the utility and limitations of the RF model in predicting nutrient distribution across varied topographies. Incorporating additional site‐specific factors, such as land use history and water table fluctuations, could improve model accuracy and better inform management of nutrient hotspots in agricultural landscapes. This framework could be extended to legacy N modeling and applied in other physiographic regions to support nutrient mitigation strategies at broader scales. In low‐relief, tile‐drained agricultural landscapes, spatial models can help identify nutrient hotspots and guide placement of mitigation practices such as buffer strips, wetlands, or drainage modifications. Incorporating field‐level management history and hydrologic context is critical for improving model accuracy and conservation outcomes.

## 4 CONCLUSIONS

This study confirmed the hypothesis that topographic and hydrologic features influence legacy P distribution and transport in low‐relief agricultural landscapes. We explored the relationships between topography, soil P distribution, and landscape properties influencing legacy P movement in a low‐relief watershed on the Delmarva Peninsula. Soil P concentrations tended to be higher in depressional areas due to gravity‐mediated transport from uplands. The legacy of near‐barn pasture use was identified as a key driver of P distribution, with hydrologic and landscape properties influencing the observed P patterns. Engineered drainage systems, including rotary ditches and tile drains, enhance P transport, and contribute to P movement through channelized overflow and tile drainage. While individual topographic parameters showed modest correlation with soil P, the RF model demonstrated complex interactions and demonstrated predictive utility for identifying CSA. By identifying critical P source areas and understanding the processes governing P transport, targeted management strategies such as controlled drainage and vegetative buffers can be implemented to reduce losses of P and other legacy nutrients such as N. These efforts can contribute to improving water quality in the Chesapeake Bay watershed.

## AUTHOR CONTRIBUTIONS

**Maryam Foroughi**: Conceptualization; data curation; formal analysis; investigation; methodology; project administration; software; visualization; writing—original draft; writing—review and editing. **Ling Du**: Conceptualization; investigation; writing—review and editing. **Isis S. P. C. Scott**: Conceptualization; investigation; methodology; writing—review and editing. **W. Dean Hively**: Conceptualization; investigation; writing—review and editing. **Zachary P. Simpson**: Conceptualization; methodology; writing—review and editing. **Zacharias J. Smith**: Methodology; writing—review and editing. **Cathleen J. Hapeman**: Conceptualization; investigation; methodology; writing—review and editing. **Martin C. Rabenhorst**: Writing—review and editing. **Raymond R. Weil**: Writing—review and editing. **Gregory W. McCarty**: Conceptualization; investigation; methodology; writing—review and editing.

## CONFLICT OF INTEREST STATEMENT

The authors declare no conflicts of interest.

## Supporting information




**Supplementary Figure 1**. The correlation between topographic parameters, soil parameters, and soil P (total P and M3‐P) in two soil depth, 0‐5 cm (A) and 5‐15 cm (B). The topographic metrics include positive openness (POP), upslope slope (UpSl), profile curvature (PCur), plan curvature (PlCur), general curvature (GCur), flow accumulation (FA), topographic relief component 1 (RePC1), topographic relief component 2 (RePC2), flow path length (FPL), catchment area (CA), topographic wetness index (TWI), stream power index (SPI), slope length factor (LS). Soil parameters (sand, silt, clay, pH, and total carbon [TC]). The color indicates direction (blue = positive, red = negative).
**Supplementary Figure 2**. The cluster correlation between infiltration and soil parameters and soil topographic metrics which include positive openness (POP), upslope slope (UpSl), Profile curvature (PCur), plan curvature (PlCur), general curvature (GCur), flow accumulation (FA), topographic relief component 1 (RePC1), topographic relief component 2 (RePC2), flow path length (FPL), catchment area (CA), topographic wetness index (TWI), stream power index (SPI), slope length factor (LS). Square size represents the magnitude of the Pearson correlation coefficient, while color indicates direction (blue = positive, red = negative).
**Supplementary Figure 3**. (A) Variable importance of soil and topographic parameters in predicting total P and M3‐P concentrations (mg kg^−1^) at the 0‐5 cm soil depth using a Random Forest model. (B) Regression plot of predicted vs. observed total P and M3‐P (mg kg^−1^) values based on the Random Forest model.
**Supplementary Figure 4**. (A) Variable importance of soil and topographic parameters in predicting total P and M3‐P concentrations (mg kg^−1^) at the 5‐15 cm soil depth using a Random Forest model. (B) Regression plot of predicted vs. observed total P and M3‐P (mg kg^−1^) values based on the Random Forest model.
**Supplementary Figure 5**. Relationship between observation and Random Forest prediction for clay %, sand %, total carbon (TC) (g kg^−1^), and infiltration (cm h^−1^) at 0‐5 cm soil depth.
**Supplementary Figure 6**. Delineation of catchment area using 2 m lidar data (Du et al., 2020), referenced to the North American Vertical Datum of 1988 (NAVD 88). Total P (mg kg^−1^) at 0‐5 cm soil depth and flow accumulation represented on the map.

**Supplementary Table 1**. Statistical description and Tukey HSD p‐values (P) for total, Mehlich III (M3) extraction, and oxalate acid extraction (OX) of sediment and soil P, Fe, Al, and Ca (mg kg^−1^), and total carbon (C) (g kg^−1^) at 0‐5 and 5‐15 cm depth.
**Supplementary Table 2**. Summary of the obtained performance result of the Random Forest model (Liaw & Wiener, 2018) using all data sets at 0‐5 cm and 5‐15 cm soil depth. Reported metrics include root mean squared error (RMSE), coefficient of determination (R^2^), and mean absolute error (MAE)

## Data Availability

All data described in this paper alongside R code to reproduce our analyses are available through Ag Data Commons: https://doi.org/10.15482/USDA.ADC/29922563.
