# Supplementary material for: Assessing the topographic distribution of legacy soil phosphorus in agricultural fields of the Delmarva Peninsula, Mid‐Atlantic Coastal Plain, USA
Source: J Environ Qual. 2025 Nov 29;55(1):e70101. doi: 10.1002/jeq2.70101 (PMC12665123; doi:10.1002/jeq2.70101)
Supplement: Supplementary file 1 — Supplementary Figure 1. The correlation between topographic parameters, soil parameters, and soil P (total P and M3‐P) in two soil depth, 0‐5 cm (A) and 5‐15 cm (B). The topographic metrics include positive openness (POP), upslope slope (UpSl), profile curvature (PCur), plan curvature (PlCur), general curvature (GCur), flow accumulation (FA), topographic relief component 1 (RePC1), topographic relief component 2 (RePC2), flow path length (FPL), catchment area (CA), topographic wetness index (TWI), stream power index (SPI), slope length factor (LS). Soil parameters (sand, silt, clay, pH, and total carbon [TC]). The color indicates direction (blue = positive, red = negative). Supplementary Figure 2. The cluster correlation between infiltration and soil parameters and soil topographic metrics which include positive openness (POP), upslope slope (UpSl), Profile curvature (PCur), plan curvature (PlCur), general curvature (GCur), flow accumulation (FA), topographic relief component 1 (RePC1), topographic relief component 2 (RePC2), flow path length (FPL), catchment area (CA), topographic wetness index (TWI), stream power index (SPI), slope length factor (LS). Square size represents the magnitude of the Pearson correlation coefficient, while color indicates direction (blue = positive, red = negative). Supplementary Figure 3. (A) Variable importance of soil and topographic parameters in predicting total P and M3‐P concentrations (mg kg−1) at the 0‐5 cm soil depth using a Random Forest model. (B) Regression plot of predicted vs. observed total P and M3‐P (mg kg−1) values based on the Random Forest model. Supplementary Figure 4. (A) Variable importance of soil and topographic parameters in predicting total P and M3‐P concentrations (mg kg−1) at the 5‐15 cm soil depth using a Random Forest model. (B) Regression plot of predicted vs. observed total P and M3‐P (mg kg−1) values based on the Random Forest model. Supplementary Figure 5. Relationship between observation and Random F [file JEQ2-55-0-s002.docx]

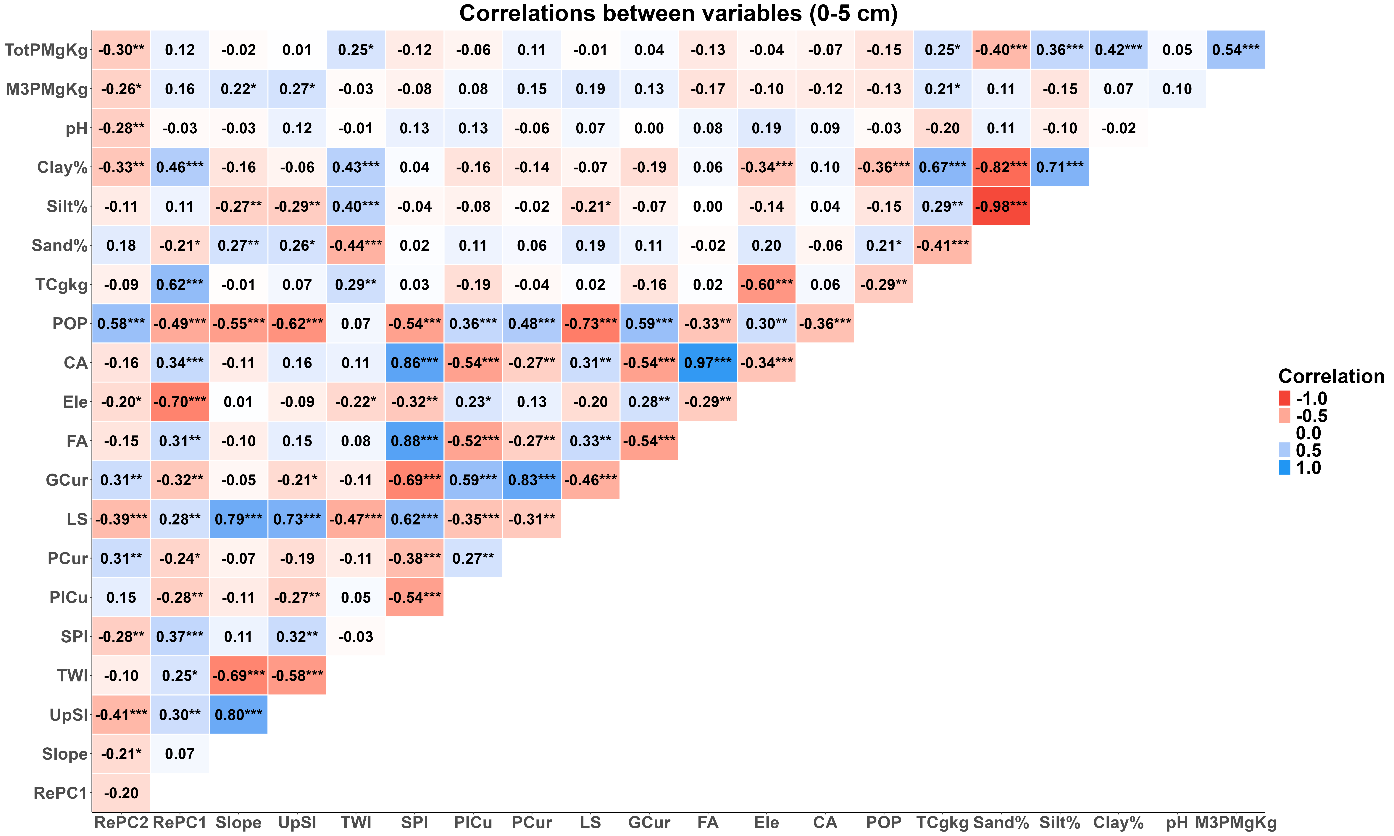

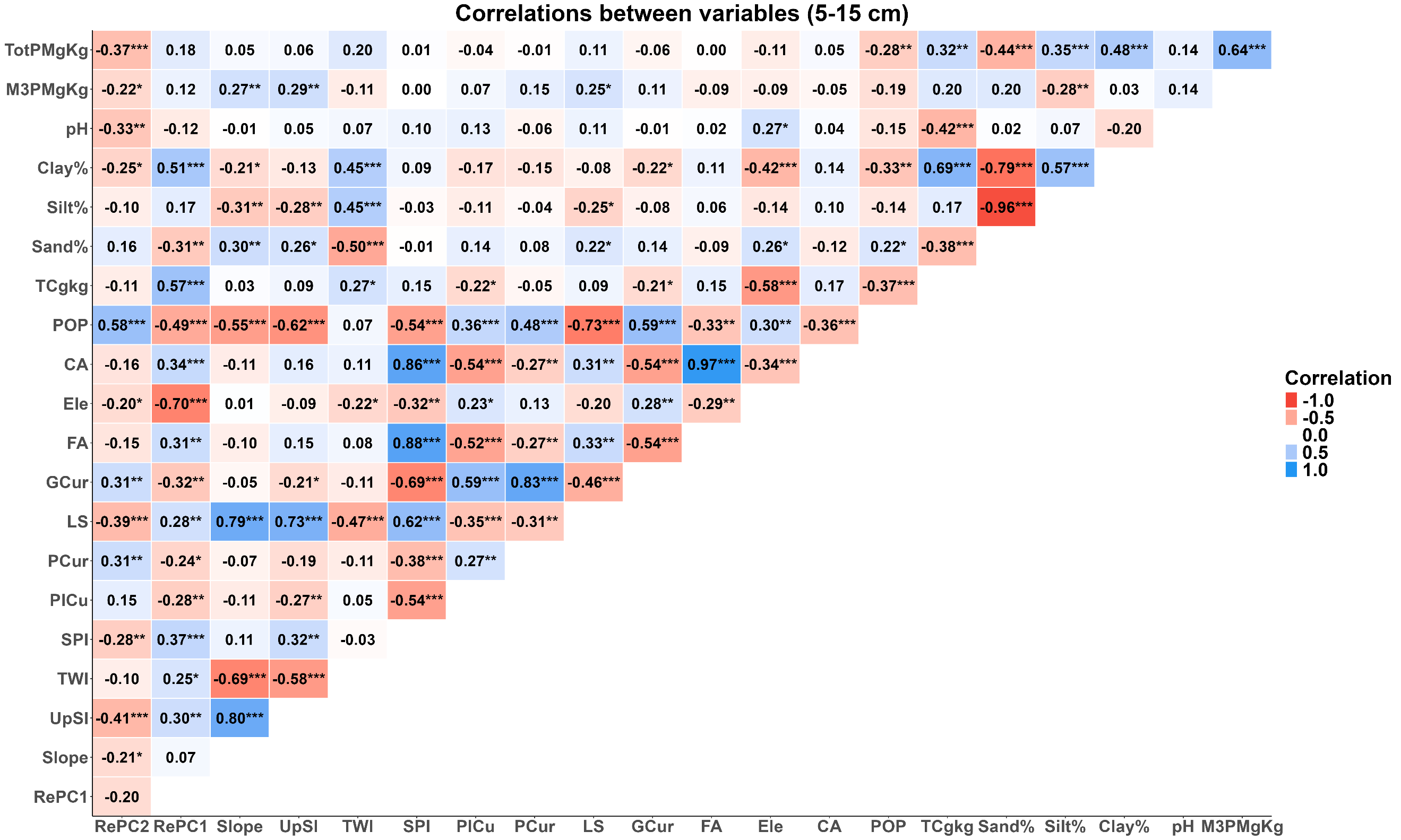


A

B

**Supplementary Figure 1.** The correlation between topographic parameters, soil parameters, and soil P (total P and M3-P) in two soil depth, 0-5 cm (A) and 5-15 cm (B). The topographic metrics include positive openness (POP), upslope slope (UpSl), profile curvature (PCur), plan curvature (PlCur), general curvature (GCur), flow accumulation (FA), topographic relief component 1 (RePC1), topographic relief component 2 (RePC2), flow path length (FPL), catchment area (CA), topographic wetness index (TWI), stream power index (SPI), slope length factor (LS). Soil parameters (sand, silt, clay, pH, and total carbon (TC)). The color indicates direction (blue = positive, red = negative).


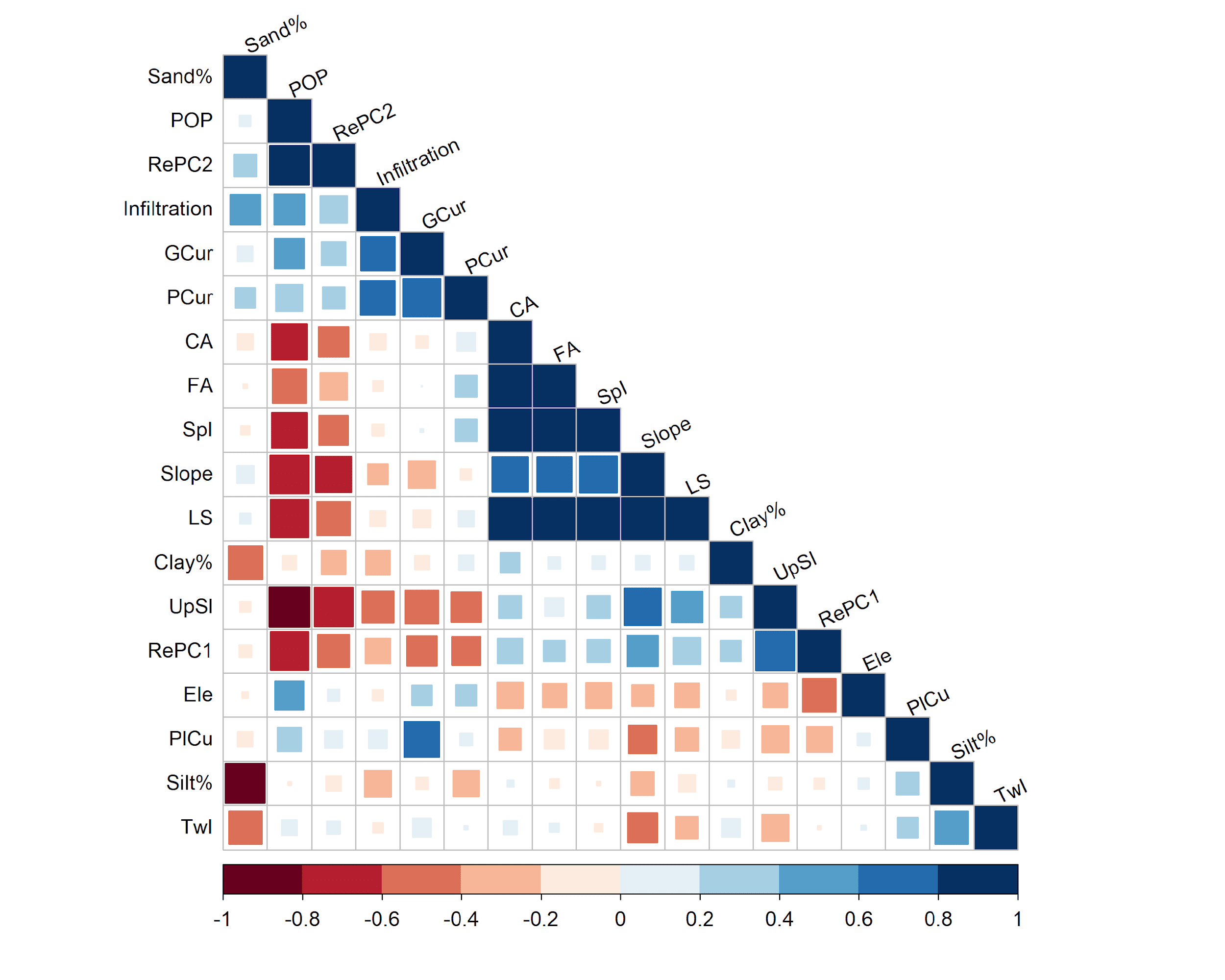


**Supplementary Figure 2.** The cluster correlation between infiltration and soil parameters and soil topographic metrics which include positive openness (POP), upslope slope (UpSl), Profile curvature (PCur), plan curvature (PlCur), general curvature (GCur), flow accumulation (FA), topographic relief component 1 (RePC1), topographic relief component 2 (RePC2), flow path length (FPL), catchment area (CA), topographic wetness index (TWI), stream power index (SPI), slope length factor (LS). Square size represents the magnitude of the Pearson correlation coefficient, while color indicates direction (blue = positive, red = negative).

**Supplementary Figure 3.** (A) Variable importance of soil and topographic parameters in predicting total P and M3-P concentrations (mg kg^-1^) at the 0-5 cm soil depth using a Random Forest model. (B) Regression plot of predicted vs. observed total P and M3-P (mg kg^-1^) values based on the Random Forest model.


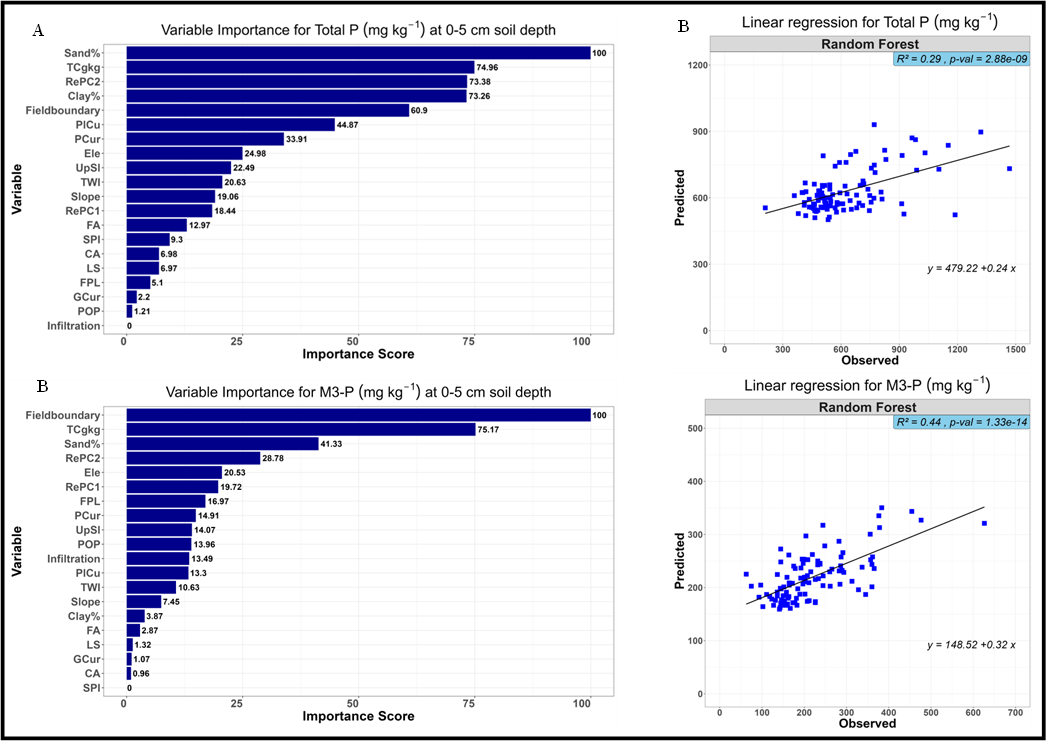


Supplementary Figure 4. (A) Variable importance of soil and topographic parameters in predicting total P and M3-P concentrations (mg kg^-1^) at the 5-15 cm soil depth using a Random Forest model. (B) Regression plot of predicted vs. observed total P and M3-P (mg kg^-1^) values based on the Random Forest model.


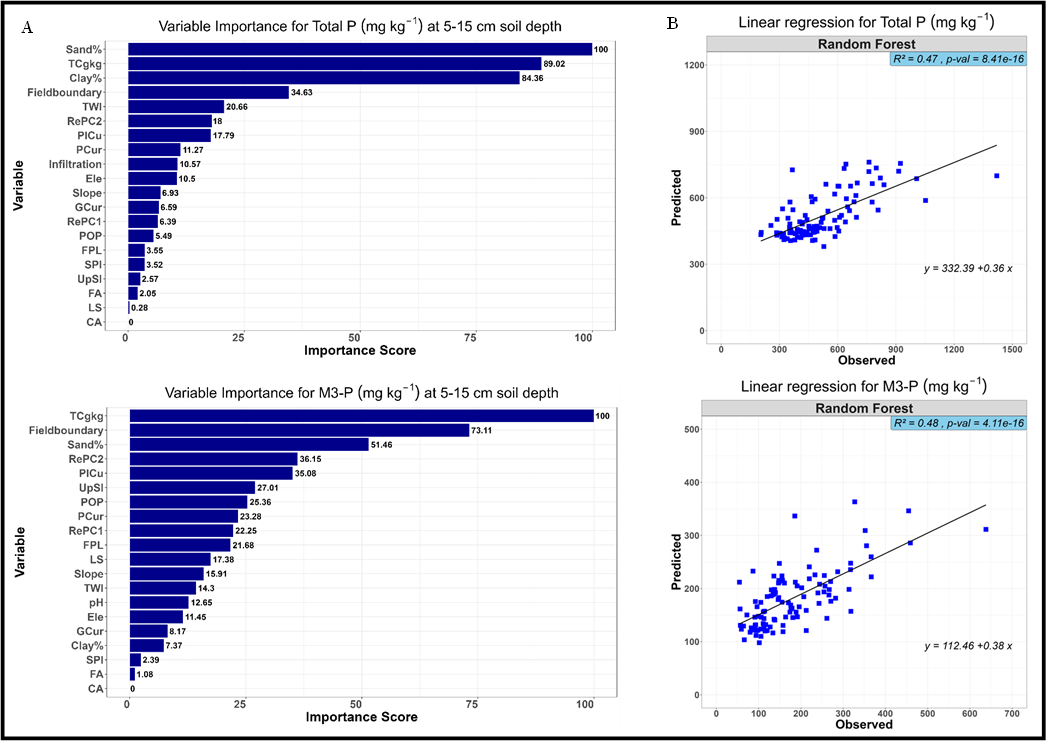

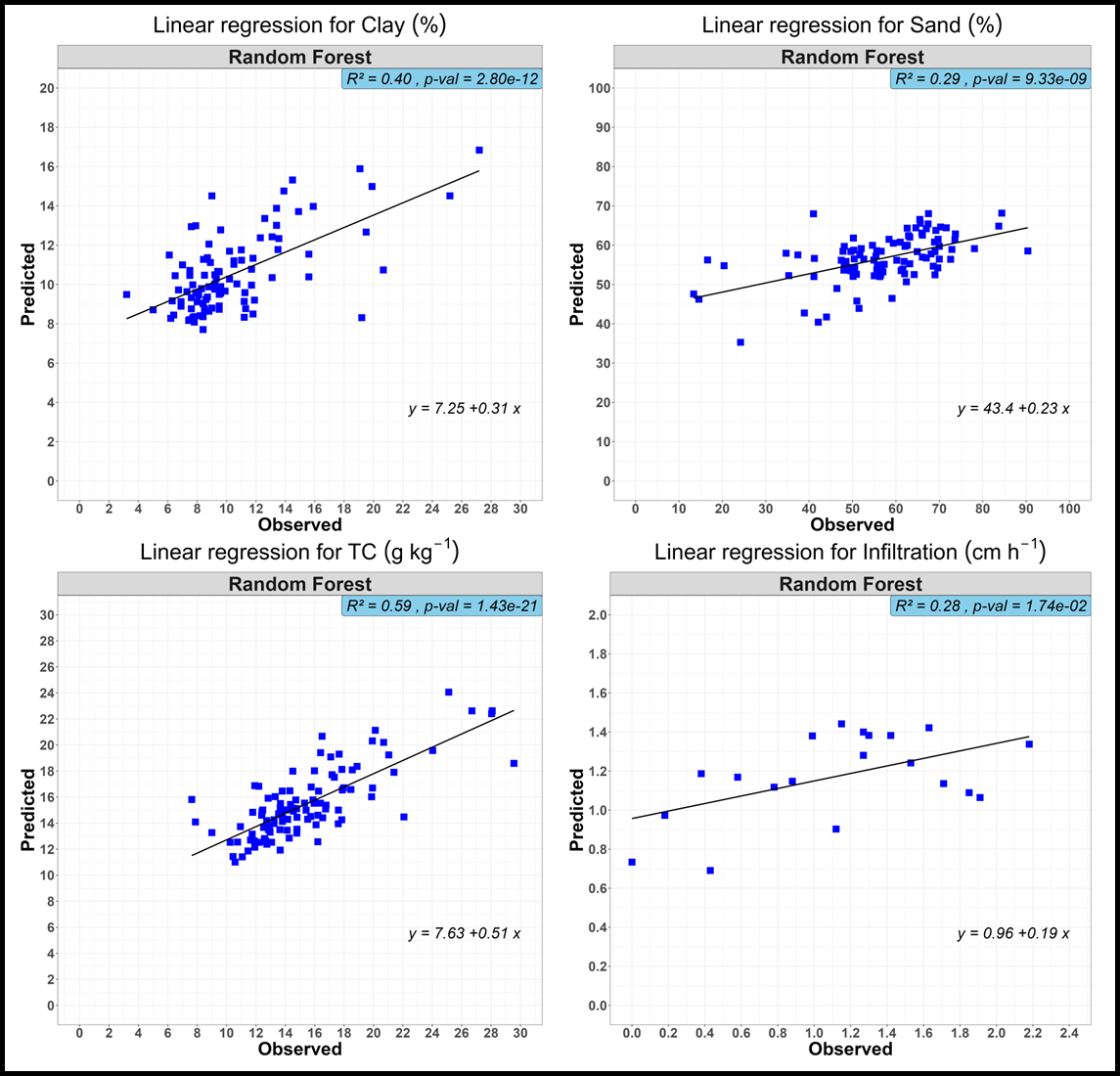


**Supplementary Figure 5.** Relationship between observation and Random Forest prediction for clay %, sand %, total carbon (TC) (g kg^-1^), and infiltration (cm h^-1^) at 0-5 cm soil depth.

**Supplementary Figure 6.** Delineation of catchment area using 2m lidar data (Du et al., 2020), referenced to the North American Vertical Datum of 1988 (NAVD 88). Total P (mg kg^-1^) at 0-5 cm soil depth and flow accumulation represented on the map.


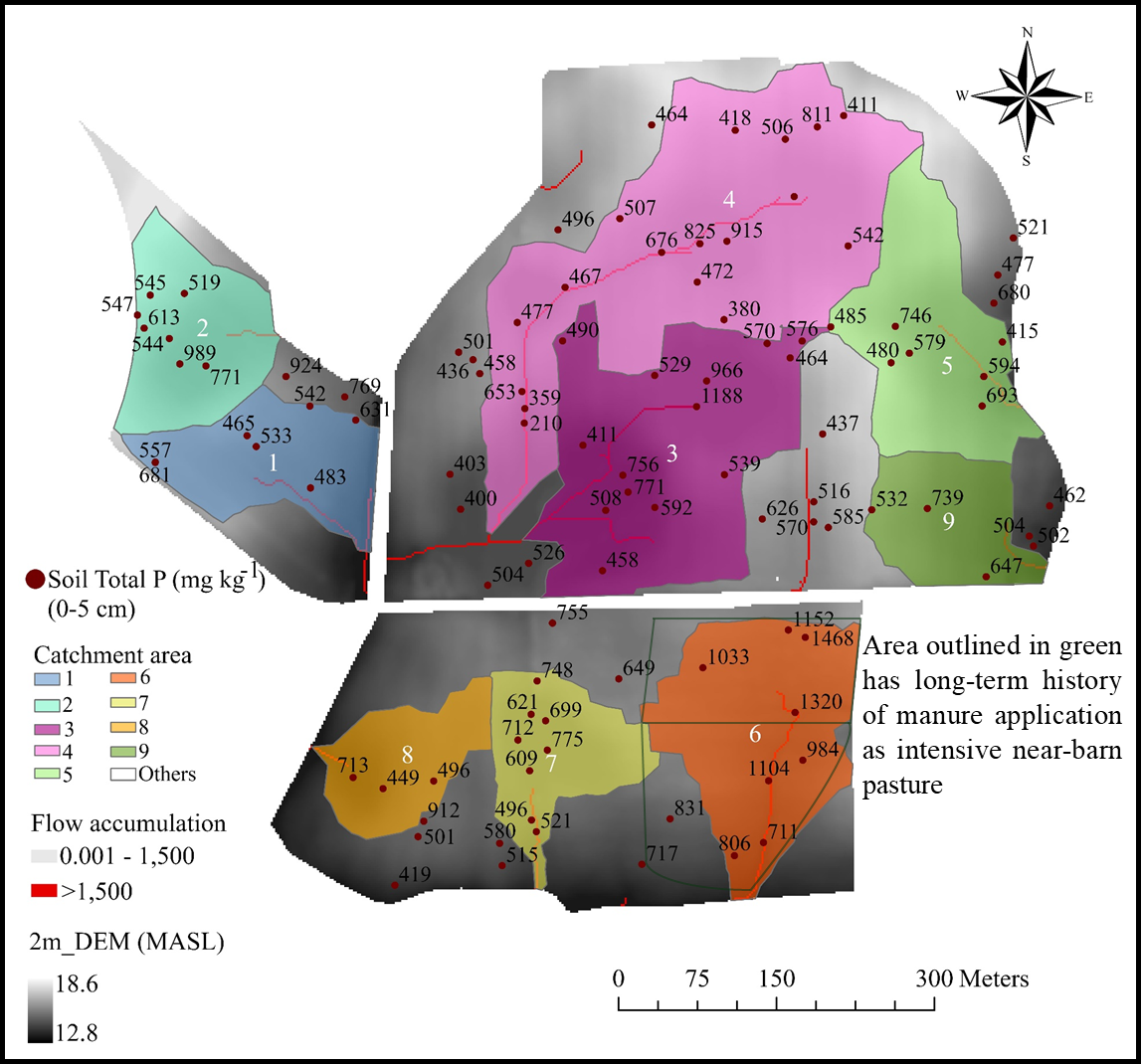


References

Du, L., McCarty, G. W., Zhang, X., Lang, M. W., Vanderhoof, M. K., Li, X., Huang, C., Lee, S., & Zou, Z. (2020). Mapping forested wetland inundation in the Delmarva Peninsula, USA using Deep convolutional neural networks. <https://www.mdpi.com/2072-4292/12/4/644>
