# Supplementary material for: Assessing the topographic distribution of legacy soil phosphorus in agricultural fields of the Delmarva Peninsula, Mid‐Atlantic Coastal Plain, USA
Source: J Environ Qual. 2025 Nov 29;55(1):e70101. doi: 10.1002/jeq2.70101 (PMC12665123; doi:10.1002/jeq2.70101)
Supplement: Supplementary file 2 — Supplementary Table 1. Statistical description and Tukey HSD p‐values (P) for total, Mehlich III (M3) extraction, and oxalate acid extraction (OX) of sediment and soil P, Fe, Al, and Ca (mg kg−1), and total carbon (C) (g kg−1) at 0‐5 and 5‐15 cm depth. Supplementary Table 2. Summary of the obtained performance result of the Random Forest model (Liaw & Wiener, 2018) using all data sets at 0‐5 cm and 5‐15 cm soil depth. Reported metrics include root mean squared error (RMSE), coefficient of determination (R2), and mean absolute error (MAE) [file JEQ2-55-0-s001.docx]

**Supplementary** **Table 1.** Statistical description and Tukey HSD p-values (P) for total, Mehlich III (M3) extraction, and oxalate acid extraction (OX) of sediment and soil P, Fe, Al, and Ca (mg kg^-1^), and total carbon (C) (g kg^-1^) at 0-5 and 5-15 cm depth.

| **Element** | **Sample No.** | **Min** | **Max** | **Mean** | **Median** | **SD** | **P: 0-5 vs 5-15** | **P: 0-5 vs sediment** | **P: 5-15 vs sediment** |  |
| --- | --- | --- | --- | --- | --- | --- | --- | --- | --- | --- |
| 0-5 cm | | | | | | | | | | |
| **Total-P** | 104 | 210 | 1.47×10^3^ | 623 | 546 | 210 | **1.24×10^-3^** | 0.98 | 0.13 |  |
| **Total-Fe** | 105 | 2.00×10^3^ | 1.37×10^4^ | 6.37×10^3^ | 6.48×10^3^ | 2.24×10^3^ | 0.96 | 0.85 | 0.92 |  |
| **Total-Al** | 105 | 4.11×10^3^ | 2.07×10^4^ | 8.50×10^3^ | 7.66×10^3^ | 3.37×10^3^ | 0.95 | **2.14×10^-6^** | 4.27**×10^-6^** |  |
| **Total-Ca** | 105 | 966 | 4.61×10^3^ | 2.11×10^3^ | 1.92×10^3^ | 768 | **2.80×10^-7^** | 0.07 | 0.89 |  |
| **M3-P** | 104 | 62.8 | 626 | 218 | 199 | 91.8 | **2.80×10^-7^** | **3.55×10^-4^** | **0.04** |  |
| **M3-Fe** | 105 | 127 | 484 | 297 | 294 | 92.0 | 0.54 | **1.40×10^-4^** | **1.59×10^-5^** |  |
| **M3-Al** | 105 | 292 | 1.07×10^3^ | 643 | 621 | 140 | 0.27 | 0.69 | 0.99 |  |
| **Ox-P** | 105 | 98.0 | 1.01×10^3^ | 389 | 351 | 147 | 0.13 | 0.66 | 0.17 |  |
| **Ox-Fe** | 105 | 374 | 2.90×10^3^ | 946 | 881 | 338 | 0.99 | **6.50×10^-12^** | **7.80×10^-12^** |  |
| **Ox-Al** | 105 | 205 | 1.30×10^3^ | 614 | 563 | 205 | 0.88 | **8.60×10^-7^** | **2.55×10^-6^** |  |
| **Total-C** | 105 | 7.63 | 29.6 | 15.4 | 14.5 | 4.05 | **4.12×10^-4^** | **3.60×10^-14^** | **3.65×10^-14^** |  |
| 5-15 cm | | | | | | | | | | |
| **Total-P** | 104 | 204 | 1.42×10^3^ | 515 | 469 | 193 |  |  |  |  |
| **Total-Fe** | 105 | 1.58×10^3^ | 1.36×10^4^ | 6.28×10^3^ | 6.29×10^3^ | 2.37×10^3^ |  |  |  |  |
| **Total-Al** | 105 | 2.44×10^3^ | 2.34×10^3^ | 8.67×10^3^ | 7.80×10^3^ | 3.74×10^3^ |  |  |  |  |
| **Total-Ca** | 105 | 562 | 4.86×10^3^ | 1.52×10^3^ | 1.32×10^3^ | 763 |  |  |  |  |
| **M3-P** | 105 | 54.6 | 638 | 179 | 149 | 98.0 |  |  |  |  |
| **M3-Fe** | 105 | 112 | 480 | 284 | 279 | 97.8 |  |  |  |  |
| **M3-Al** | 105 | 460 | 1.14×10^3^ | 672 | 655 | 138 |  |  |  |  |
| **Ox-P** | 105 | 128 | 984 | 345 | 303 | 155 |  |  |  |  |
| **Ox-Fe** | 105 | 425 | 3.06×10^3^ | 951 | 864 | 382 |  |  |  |  |
| **Ox-Al** | 105 | 344 | 1.36×10^3^ | 631 | 578 | 214 |  |  |  |  |
| **Total-C** | 105 | 6.55 | 25.0 | 11.8 | 10.7 | 3.90 |  |  |  |  |
| Sediment | | | | | | | | | | |
| **Total-P** | 14 | 177 | 1.53×10^3^ | 636 | 445 | 390 |  |  |  |  |
| **Total-Fe** | 14 | 1.82×10^3^ | 1.19×10^4^ | 6.02×10^3^ | 5.25×10^3^ | 2.73×10^3^ |  |  |  |  |
| **Total-Al** | 14 | 4.69×10^3^ | 3.07×10^4^ | 1.41×10^4^ | 1.25×10^4^ | 7.21×10^3^ |  |  |  |  |
| **Total-Ca** | 14 | 546 | 3.61×10^3^ | 1.62×10^3^ | 1.48×10^3^ | 859 |  |  |  |  |
| **M3-P** | 14 | 55.1 | 360 | 113 | 88.5 | 77.6 |  |  |  |  |
| **M3-Fe** | 14 | 436 | 6.88×10^3^ | 2.12×10^3^ | 1.44×10^3^ | 1.78×10^3^ |  |  |  |  |
| **M3-Al** | 14 | 385 | 1.51×10^3^ | 999 | 813 | 638 |  |  |  |  |
| **Ox-P** | 14 | 88.4 | 1.24×10^3^ | 430 | 279 | 322 |  |  |  |  |
| **Ox-Fe** | 14 | 436 | 6.88×10^3^ | 2.12×10^3^ | 1.44×10^3^ | 1.78×10^3^ |  |  |  |  |
| **Ox-Al** | 14 | 385 | 1.51×10^3^ | 999 | 813 | 638 |  |  |  |  |
| **Total-C** | 14 | 15.8 | 95.7 | 44.0 | 36.5 | 22.5 |  |  |  |  |

*Note*: The post hoc *p*-value was conducted using Tukey’s HSD test. P-values less than 0.05 were considered statistically significant and are shown in bold.

**Supplementary** **Table 2.** Summary of the obtained performance result of the Random Forest model (Liaw & Wiener, 2018) using all data sets at 0-5 cm and 5-15 cm soil depth. Reported metrics include root mean squared error (RMSE), coefficient of determination (R2), and mean absolute error (MAE)

|  | **RMSE** | ***R^2^*** | **MAE** |
| --- | --- | --- | --- |
| **0-5 cm** | | | |
| Clay % | 3.12 | 0.40 | 2.10 |
| Sand % | 11.8 | 0.29 | 8.60 |
| Total C (g kg^-1^) | 2.62 | 0.59 | 1.84 |
| Infiltration | 0.20 | 0.28 | 0.17 |
| M3-P (mg kg^-1^) | 70.1 | 0.44 | 52.4 |
| Total P (mg kg^-1^) | 177 | 0.29 | 131 |
| **5-15 cm** | | | |
| Clay % | 3.81 | 0.38 | 2.66 |
| Sand % | 11.18 | 0.31 | 8.51 |
| Total C (g kg^-1^) | 2.67 | 0.54 | 1.95 |
| M3-P (mg kg^-1^) | 71.8 | 0.48 | 54.5 |
| Total P (mg kg^-1^) | 100 | 0.47 | 143 |

References

Liaw, A., & Wiener, M. (2018). Package “Random Forests.”https:// www. stat. berkeley. edu/ %7 Ebreiman/RandomForests/
